# Supplementary material for: Two-step offer and return of multiple types of additional genomic findings to families after ultrarapid trio genomic testing in the acute care setting: a study protocol
Source: BMJ Open. 2023 Jun 2;13(6):e072999. doi: 10.1136/bmjopen-2023-072999 (PMC10254691; doi:10.1136/bmjopen-2023-072999)
Supplement: Author's manuscript [file bmjopen-2023-072999.draft_revisions.pdf]

BMJ Open is committed to open peer review. As part of this commitment we make the peer review history of every article we publish publicly available.

When an article is published we post the peer reviewers' comments and the authors' responses online. We also post the versions of the paper that were used during peer review. These are the versions that the peer review comments apply to.

The versions of the paper that follow are the versions that were submitted during the peer review process. They are not the versions of record or the final published versions. They should not be cited or distributed as the published version of this manuscript.

BMJ Open is an open access journal and the full, final, typeset and author-corrected version of record of the manuscript is available on our site with no access controls, subscription charges or pay-per-view fees (http://bmjopen.bmj.com).

If you have any questions on BMJ Open's open peer review process please email info.bmjopen@bmj.com

BMJ Open

A study protocol for the two-step offer and return of multiple types of additional genomic findings to families after ultra-rapid trio genomic testing in the acute care setting

Journal:	*BMJ Open*
Manuscript ID	bmjopen-2023-072999
Article Type:	Protocol
Date Submitted by the Author:	20-Feb-2023

Complete List of Authors:	Bouffler, Sophie; Australian Genomics Health Alliance,<br>Lee, Ling; Walter and Eliza Hall Institute of Medical Research, Melbourne Genomics Health Alliance ; Murdoch Children's Research Institute<br>Lynch, Fiona; Murdoch Children's Research Institute; The University of Melbourne<br>Martyn, Melissa; Walter and Eliza Hall Institute of Medical Research, Melbourne Genomics Health Alliance ; Murdoch Children's Research Institute<br>Lynch, Elly; Walter and Eliza Hall Institute of Medical Research, Melbourne Genomics Health Alliance ; Victorian Clinical Genetics Services Ltd<br>Macciocca, Ivan; The University of Melbourne; Victorian Clinical Genetics Services Ltd<br>Curnow, Lisette; Victorian Clinical Genetics Services Ltd<br>McCorkell, Giulia; Australian Genomics Health Alliance; The University of Melbourne<br>Lunke, Sebastian; Victorian Clinical Genetics Services Ltd; The University of Melbourne<br>Chong, Belinda; Victorian Clinical Genetics Services Ltd<br>Marum, Justine E; Victorian Clinical Genetics Services Ltd<br>Delatycki, Martin; Victorian Clinical Genetics Services Ltd; The University of Melbourne<br>Downie, Lilian; Murdoch Children's Research Institute; The University of Melbourne<br>Goranitis, I; The University of Melbourne; Australian Genomics Health Alliance<br>Vears, Danya F; Murdoch Children's Research Institute; The University of Melbourne<br>Best, Stephanie; Australian Genomics Health Alliance; Peter MacCallum Cancer Centre<br>Clausen, Marc; Unity Health Toronto, Genomics Health Services Research Program, St. Michael's Hospital<br>Bombard, Yvonne; Unity Health Toronto, Genomics Health Services Research Program, St. Michael's Hospital; University of Toronto Institute of Health Policy Management and Evaluation<br>Stark, Zornitza; Australian Genomics Health Alliance; Victorian Clinical Genetics Services Ltd<br>Gaff , Clara; Walter and Eliza Hall Institute of Medical Research,

	Melbourne Genomics Health Alliance ; The University of Melbourne
Keywords:	GENETICS, Paediatric intensive & critical care < PAEDIATRICS, Protocols & guidelines < HEALTH SERVICES ADMINISTRATION & MANAGEMENT, Neonatal intensive & critical care < INTENSIVE & CRITICAL CARE, PAEDIATRICS, QUALITATIVE RESEARCH

A study protocol for the two-step offer and return of multiple types of additional genomic findings to families after ultra-rapid trio genomic testing in the acute care setting

Sophie E Bouffler[1], Ling Lee[2,3], Fiona Lynch[3,4], Melissa Martyn[2,3], Elly Lynch[2,5], Ivan Macciocca[4,5], Lisette Curnow[5], Giulia McCorkell[1,4], Sebastian Lunke[4,5], Belinda Chong[5], Justine E Marum[5], Martin Delatycki[4,5], Lilian Downie[3,5], Ilias Goranitis[1,6], Danya F Vears[3,4], Stephanie Best[1,7], Marc Clausen[8], Yvonne Bombard[8,9], Zornitza Stark[1,5], Clara L Gaff[2,3]

[1]Australian Genomics, Melbourne, Australia

[2]Melbourne Genomics Health Alliance, Melbourne, VIC, Australia

[3]Murdoch Children's Research Institute, Melbourne, VIC, Australia

[4]University of Melbourne, Melbourne, Australia

[5]Victorian Clinical Genetics Services, Murdoch Children's Research Institute, Melbourne, Australia

[6]Health Economics Unit, Centre for Health Policy, Melbourne School of Population and Global Health, University of Melbourne, Melbourne, Australia

[7]Peter MacCallum Cancer Centre, Melbourne, Australia

[8] Genomics Health Services Research Program, St. Michael's Hospital, Unity Health Toronto, Toronto, ON, Canada

[9]Institute of Health Policy, Management and Evaluation, University of Toronto, Toronto, ON, Canada

For peer review only

Corresponding author: Clara Gaff, clara.gaff@melbournegenomics.org.au, Melbourne Genomics Health Alliance, Melbourne, VIC, Australia, Murdoch Children's Research Institute, Melbourne, VIC, Australia

Word count: 3256

Keywords: Additional findings, genomics, acute care, decision support

For peer review only

ABSTRACT

Introduction As routine genomic testing expands, so too does the opportunity to look for additional health information unrelated to the original reason for testing, termed additional findings (AF). Analysis for many different types of AF may be available, particularly to families undergoing trio genomic testing. The optimal model for service delivery remains to be determined, especially when the original test occurs in the acute care setting.

Methods and analysis Families enrolled in a national study providing ultra-rapid genomic testing to critically ill children will be offered analysis for three types of AF on their stored genomic data: paediatric-onset conditions in the child, adult-onset conditions in each parent, and reproductive carrier screening for the parents as a couple. The offer will be made three- to six-months after diagnostic testing. Parents will have access to a modified version of the Genetics Adviser web-based decision support tool before attending a genetic counselling appointment to discuss consent for AF. Parental experiences will be evaluated using qualitative and quantitative methods on data collected through surveys, appointment recordings, and interviews at multiple timepoints. Genetic health professionals' perspectives on acceptability and feasibility of AF will also be captured through surveys and interviews.

Ethics and dissemination This project received ethics approval from the Melbourne Health Human Research Ethics Committee as part of the Australian Genomics Health Alliance protocol: HREC/16/MH/251. Findings will be disseminated through peer-review journal articles and at conferences nationally and internationally.

ARTICLE SUMMARY

Strengths and limitations of this study

- This is a prospective national pilot study across 17 clinical sites exploring the offer and receipt of three types of AF following genomic testing in the acute care setting.
- This study will investigate the practicability of offering AF analysis to parents and children using stored genomic data 3-6 months after diagnostic testing.
- Results from this study can inform future health system implementation of analysis for AF.
- A process evaluation will be undertaken to investigate parental decision-making and preferences for health service delivery.
- This study explores AF specifically in the context of acute neonatal and paediatric care.

INTRODUCTION

Additional findings (AF) refer to results from genomic testing that may have medical value and/or utility and are found in a deliberate search unrelated to the original reason for testing.[1,2] Such findings may include adult- and paediatric-onset treatable and untreatable disorders, reproductive carrier status and pharmacogenomic testing. Research participants consistently express a high level of interest in receiving AF.[3-5]

For peer review only

The American College of Medical Genetics and Genomics (ACMG) initially advocated the return of adult-onset AF as a mandatory part of genomic testing regardless of the patient's age,[1] with updated guidelines suggesting an opt-in approach with a minimum list of 73 genes to be reported.[6] The offer of paediatric-onset AF for children undergoing genomic sequencing has been explored, often as a prelude to developing newborn screening programs.[7,8] Stored genomic data can also be utilised for reproductive genetic carrier screening. As carrier screening becomes more common[9] it is a natural extension for parents undergoing trio genomic testing to be offered this additional information as they are typically of child-bearing age. A recent systematic review[10] summarised the position of several countries, with many international bodies, including the Canadian College of Medical Geneticists and the European Society of Human Genetics (ESHG), supporting a more conservative approach and recommended that analysis and reporting from genomic sequencing should be focused on the primary reason for testing.[11,12] In Australia, analysis is largely restricted to genes known to be associated with the patient's primary clinical indication for testing.

Determining whether and how to deliver AF within health services continues to be debated by professional bodies. International studies have described models whereby the offer of AF is made at the time diagnostic testing is initiated.[13-15] There are concerns that this model adds to the complexity of pre-test counselling at a time when families are already overwhelmed by having to make many complex decisions. This is particularly pertinent in the acute neonatal and paediatric care setting, when parents are dealing with decision-making for their critically ill child in a highly stressful, time pressured environment. Concerns

For peer review only

have been raised about parents' ability to process and retain information and the impact of genomic results on family functioning in this setting.[16-21] Emerging evidence supports the validity of these concerns,[22] and in one study the offer of AF deterred families from receiving genetic testing when a diagnostic result would be in the child's best interest.[23] The ESHG have more recently acknowledged the method proposed by the French Society of Predictive and Personalized Medicine, which recommends a multi-step approach to consent for AF,[24] but note the need for further empirical evidence.[25]

The Melbourne Genomics Health Alliance described a pilot study trialling a two-step offer for AF analysis on stored data in adult patients, with the offer occurring subsequent to the primary result return.[26] This model allows for temporal separation of the offer and avoids burdening patients with complex decisions at a time of high stress, while still offering them the opportunity to receive more health information from their stored genomic data.

The routine provision of AF in a two-step model could involve a significant investment of time and resources.[27] As evidence reinforcing the importance of providing detailed information during the informed consent process in this space emerges[28] it is essential to explore tools that will support this. Digital decision support tools have the potential to alleviate some of the resource constraints when implemented in a genomic care pathway, alongside in-person encounters.[29,30]

As genomic sequencing is increasingly incorporated into different clinical settings there continues to be a need for evidence to inform decisions about how healthcare systems

For peer review only

should manage AF. Storage of genomic data enables analysis for AF as a separate decision after diagnostic testing is complete.

The Acute Care Genomics (ACG) study[31] is evaluating ultra-rapid trio whole genome sequencing for critically ill infants and children on a national scale. The study cohort includes parents and children who have stored whole genome sequencing data from trio testing. This cohort provides the opportunity to understand the service implications of offering and returning multiple AF in acute care on a national scale, as well as parental preferences for receiving this information, across multiple genetic services.

Study aim

We describe a prospective national study offering analysis for multiple types of additional findings on stored data to children and their parents undergoing trio genomic testing via a two-step delivery model. This study uses process evaluation to examine decision-making, decision support, counselling processes, as well as resource and service implications.

METHODS AND ANALYSIS

Eligibility and recruitment

This study will recruit families via a two-step model as described in a previous study.[26] The ACG study will offer up to 250 families ultra-rapid genomic trio/duo testing at 17 clinical sites around Australia between July 2020 and April 2022. As part of their participation in the ACG study, parents will be approached three- to six-months after disclosure of the child's ultra-rapid genomic testing result to be offered analysis for AF using their stored genomic data. To reflect possible clinical scenarios, parents who enquire seeking recurrence or

For peer review only

reproductive risk information before the study-prompted offer of AF may have the opportunity for earlier analysis. This study will run from July 2020 to June 2023. Study data will be managed using REDCap electronic data capture tools hosted at the Murdoch Children's Research Institute, Melbourne, Australia.[32,33]

Families will be excluded where the child is still a hospital inpatient, or if the proband's death is under coroner's investigation. Under exceptional circumstances, if the referring clinical team deem it inappropriate to approach a family for psychosocial reasons they will be excluded.

Families will be eligible to receive three types of AF:

1. Paediatric-onset additional findings in the child
2. Adult-onset additional findings in each parent
3. Expanded couple genetic carrier screening

Where the child is deceased, paediatric-onset AF will not be offered but testing will be offered to the parents. Expanded couple genetic carrier screening will not be offered for duos (one parent and child), or where the parents are separated.

Study design

The study design is outlined in Figure 1.

To reflect expected real-world service delivery and facilitate continuity of care, families will be approached by the original referring clinical service and the same provider who saw

For peer review only

them for the initial testing where feasible. Alternatively, the study team will send an approach letter via email including a link to a survey asking parents for their expression of interest. Parents will be approached in separate emails if the referring team so advise.

Parents who express interest will receive login details for a web-based decision support tool and a copy of the patient information sheet. The counselling team will subsequently contact families to arrange a pre-test genetic counselling appointment to discuss consent. All families must meet with a genetic health professional to proceed with receiving AF.

Decision support

The study will deploy an interactive web-based decision support tool as an adjunct to a genetic counselling appointment, called Genetics Adviser.[34] The tool is an expanded version of the Genomics ADvISER, which has been shown to effectively augment genetic counselling and improve patient knowledge and decisional confidence.[30,35] The Genetics Adviser is a comprehensive, interactive, patient-centred platform aimed at providing lay language digital content to support informed decision making, offer pre- and post- genetic testing support, and facilitate return of results. The tool can be tailored for particular use cases as well as a range of clinical and population testing scenarios.[34] For this protocol, the Genetics Adviser was customised to focus on pre-test counselling and education for AF with the support of genetic counselling in this process.[36] Content has been adapted in collaboration with the Genetics Adviser team to enable the delivery of tailored information on the three types of AF offered in this study with a focus on using plain language for the content and videos.

For peer review only

Each parent will receive separate login details for the Genetics Adviser and will be encouraged to work through the tool individually prior to the pre-test genetic counselling appointment. An alternative version of the tool will be made for families where the child has died, which does not contain the section on paediatric-onset additional findings.

Genetic counselling and consent

All families will receive genetic counselling. Pre- and post-test genetic counselling appointments will be conducted in person or via telehealth. Families will be asked for consent to audio record pre- and post-test genetic counselling appointments following an approved verbal consent script.

Genetic health professionals who will conduct pre-test counselling for AF will be invited to an online education workshop facilitated by genetic counsellors with experience delivering AF in a research setting. The workshop will include lessons from previous studies offering AF, small group role plays of AF counselling scenarios, and detailed study logistics. There will be a pre-workshop self-directed module including two video recorded simulation pre-test counselling appointments using trained counsellors and actors. The participating genetic health professionals will be provided with talking points and tips for counselling families to ensure that the information given and language used is consistent between services, as well as a visual aide summarising the different types of AF (Figure 2).

All participants will provide informed consent for data collection as part of their involvement in the Acute Care Genomics study.[31] This consent encompasses the research

For peer review only

component of the AF study, with parents providing separate clinical consent to the different types of AF.

Clinical consent for AF will be captured via the REDCap e-consent framework.[37] For the majority of families, consent will be documented using a single clinical e-consent form that displays all of the AF offerings the family are eligible for. Where the referring team indicate the decision for adult-onset AF will be made in separate appointments, the genetic health professional will have access to individual e-consent forms for each parent. Consent from one parent is required to proceed with analysis for paediatric-onset AF in the child; however, the genetic health professional will discuss this decision with both parents and ensure there is a consensus where possible as per standard clinical practice. Both parents must provide consent for couple genetic carrier screening to proceed.

All results will be returned by a genetic health professional in person or via telehealth/phone. All results will be returned simultaneously, unless the couple are pregnant and have requested carrier testing, in which case the results may be returned early to facilitate reproductive planning.

Gene lists

Gene lists have been created on PanelApp Australia (https://panelapp.agha.umccr.org/)[38,39] to guide analysis for the different types of AF. The content on this platform is openly available.

For peer review only

The gene list for the Australian Reproductive Carrier Screening Project (Mackenzie's Mission_Reproductive Carrier Screening) will be used for couple carrier screening.[40] Paediatric-onset additional findings (Additional findings_Paediatric) will encompass genes tested as part of the BabySeq project,[41] NC NEXUS study,[42] and Baby Beyond Hearing project.[7] The final list includes treatable and untreatable conditions affecting children. The gene list for adult-onset additional findings (Additional findings_Adult) is based on the list created for the Melbourne Genomics Additional Findings flagship,[26] which includes genes associated with clinically actionable conditions where a publicly funded management pathway exists. This list was updated to include genes on the ACMG V3.0 Secondary Findings list.[6]

Laboratory protocol

The laboratory will receive separate requests for each type of AF to which a family has given consent. The laboratory will analyse the stored genomic data according to National Association of Testing Authorities (NATA) clinically accredited procedures. Variants will be classified based on ACMG classification guidelines[43] and only pathogenic or likely pathogenic variants will be reported. All variants identified for reporting will be discussed in a multidisciplinary team meeting, including clinical geneticists, other relevant medical subspecialists, genetic counsellors, bioinformaticians, and molecular geneticists. Variants of uncertain significance and individual carrier results will not be reported. The standard turnaround time for AF reports will be 12 weeks. Reproductive carrier screening results will be expedited if clinically indicated.

For peer review only

Where the child has previously been diagnosed with an autosomal recessive, X-linked or inherited dominant condition, increased reproductive risk will be included on the couple carrier screening report, regardless of whether the gene is contained in the carrier screening panel.

Evaluation

This study will answer the following questions:

1. What is the uptake of the different types of AF?
2. What influences people's decisions to accept or decline the different types of AF?
3. How is the decision support tool used?
4. What are stakeholder perspectives of the decision support tool?
5. To what extent do counselling tools and processes support understanding of AF?
6. What are stakeholders' (patients and genetic health professionals) views of offering AF in the acute care setting?

Clinical and genetic counselling data collection

We will collect data on how many families are offered, accept, and decline AF analysis and at which stage of the process this occurs. We will collect data on the types of AF families choose to have and the outcomes of analysis. Data will also be collected on laboratory turnaround times. Pre- and post-test genetic counselling appointments will be audio recorded.

Surveys: Parents

For peer review only

Participants will be invited to prospectively complete surveys throughout the study (see Figure 1). All surveys will be delivered electronically to each parent via the study's REDCap database. Participants will be invited to complete the first survey (T1) prior to the pre-test counselling appointment, the second (T2) after deciding whether to accept any or decline all AF and the third (T3) following result return. If participants decline before counselling, they will receive a decliner survey (T0); immediately for active decliners (parents who explicitly define AF) or six months after approach for passive decliners (parents who do not respond to contact from the clinical or study team).

Survey measures are outlined in

Table 1. T1 will capture perspectives on the decision tool, parent understanding of the AF offer, and baseline psychometrics. T2 will evaluate processes and reasons for decision-making as well as parental values and understanding of analysis for AF post-counselling. T3 will capture information on the participant's values, experience and understanding of AF results.

For peer review only

Table 1: Survey measures

**Measure**	**Description**	**T1**	**T2**	**T3**	**T0**
Decision support tool user assessment	Six study-specific questions about decision support use, clarity, relevance, bias, other information needs and areas of improvement.	X			
Knowledge of AF	Seven study-specific questions to determine a participants' understanding of AF offered.	X	X		
State trait anxiety index (STAI-AD)	26-item scale measuring state and trait anxiety. *Copyright © 1968, 1977 by Charles D. Spielberger. All rights reserved in all media. Published by Mind Garden,Inc.* *www.mindgarden.com*	X	X	X	X

	Note: The 6 questions that form the STAI-6 short form[44] are repeated at each timepoint. If participants complete any of the remaining 20 items in any survey they will not be asked these items again.				
Health behaviour	A shortened version of the Threatening Medical Situations Inventory.[45-47]	X	X		X
Demographics	Age, gender, education, income, language, marital status, number of children, prior experience with genomic testing, family planning, private health insurance status, postcode.<br>Note: if participants complete demographic questions in any survey they will not be asked again.	X	X	X	X
Decision recall	One study-specific question to assess whether parents recall their decision.		X	X	
Difficulty and deliberation of decision	Two study-specific questions to assess how difficult it was and how long it took parents to decide which AF to receive (T2) or to decline (T0)		X		X
Reasons for accepting or declining	Three study-specific questions for each type of AF offered addressing reasons for the participant's decision. Participants are asked to rate a selection of reasons on a 5-point Likert scale and asked to comment if there are other reasons not listed.<br>Note: separate questions for acceptors and decliners of the different types of AF		X		
Decisional conflict scale	16-item scale measuring decisional conflict.[48]		X		
Acceptable information needs	Two study-specific questions about whether use of the decision tool alone without genetic counselling would provide enough information to make a decision.		X		
Genetic counselling satisfaction	Seven-item scale addressing patient satisfaction and service quality in the clinical genetics setting.[49]		X	X	
Willingness to pay	Dynamic triple-bounded dichotomous choice contingent valuation, also known as a 'bidding game' to assess the value participants place on the information that comes from analysis for AF.		X	X	
Results recall and understanding of results	Four study-specific questions to determine if participants recalled their results correctly, and if they understand what these results mean.			X	

For peer review only

Future planning	Five study-specific questions on future planning for themselves, their child, and their family based on the AF results received.			X	
Decision regret scale	Five-item scale to measure regret after a health care decision.[50]			X	
FACToR questionnaire	12-item questionnaire adapted to assess the psychosocial impact of returning genomic test results.[51]			X	
Genomics outcome scale (GOS)	Six-item scale based on the Genetic Counselling Outcome Scale (GCOS-24) adapted to assess outcomes of genetic counselling and patient empowerment.[52]			X	
Service delivery preferences	Three study-specific questions addressing how and when the offer for AF should be made.			X	
Value of offering AF	One study-specific question on the value of information that comes from analysis for AF. Participants are asked to rate different factors on a 4-point Likert scale from extremely valuable to not valuable.			X	
Reasons for declining	One study-specific question exploring the reasons for declining. Participants are presented with 13 reasons and asked to rate how much each one influenced their decision on a scale of 1 (did not influence) to 5 (strongly influenced).				X

AF – additional findings, T1 – pre-test counselling survey, T2 – post-decision for AF survey, T3 – post-result return survey, T0 – passive or active decliner survey

For peer review only

Parent interviews

Participants will be invited to provide their contact details during any of the surveys if they are willing to be contacted for an interview to discuss their experience of the study in greater detail. Those who do so will be offered the option to participate in a semi-structured interview, either by phone or via an online platform. The interviews will explore participants' experiences with the web-based decision support tool, genetic counselling appointment, consent process, and result disclosure. Interviews will be audio-recorded, transcribed, deidentified, and analysed using inductive content analysis.

Genetic health professional evaluation

The perspectives of genetic health professionals (genetic counsellors and clinical geneticists) implementing AF in this study will be evaluated using a mixed methods study. Participating health professionals will be identified by the study team and receive a validated survey[53] prior to commencing the AF study with families and upon completion of result return to the final participants. The survey aims to establish practitioners' perspectives of the implementation outcomes of acceptability, appropriateness, and feasibility.[54] Follow up semi-structured interviews will be undertaken through an online platform to investigate influences on clinicians' perspectives and to identify enablers to the adoption of AF for practitioners. Transcripts will be audio recorded and transcribed before deductive and inductive data analysis is undertaken.

Data analysis

Overall AF uptake will be reported as the number of families who accept any type of AF divided by the total offered any AF. Uptake of each type of AF will also be reported, noting that the denominator for each AF offer may differ.

Quantitative data from surveys (Likert-scales, categorical items) will be analysed using standard statistical methodology. If sample sizes for each group provide sufficient power, multivariate statistical analyses will be performed to explore factors that may influence uptake (e.g., received primary diagnosis or not, family completion status). Economic implications associated with the delivery of AF and the effect on follow-on care pathways will be evaluated using health economic modelling methods, subject to group size.

For peer review only

Quantitative data on decision support tool uptake and utilisation will be analysed. Uptake will be measured by determining the number of parents who accessed the Genetics Adviser divided by the number of parents who were invited. Utilisation will be measured by the time parents took to work through the Genetics Adviser and the drop off rate.

Qualitative data will include open-text survey responses and audio recordings (of interviews and genetic counselling sessions). Open-text survey responses will be analysed iteratively, using inductive content analysis, and used to explain the quantitative survey findings. The approach to analysis of counselling transcripts will be informed by responses to surveys and decision support tool utilisation metrics. It is anticipated that a hybrid deductive-inductive content analysis approach will be used to code counselling interactions to explore the impact of use of the decision support tool on counselling and decision making and identify counselling challenges (specific to the acute care setting).

Patient and Public Involvement

The design and delivery of this study has been heavily informed by previous consumer focused studies,[7,26] incorporating learnings from participant responses and feedback gathered in these studies. The study also uses Genetics Adviser, a decision support platform with extensive public input into design and development.[30,34-36]

DISCUSSION

This protocol describes the offer and return of three categories of AF to families participating in a study examining national implementation of ultra-rapid genomic diagnostic testing in the acute care setting. We will utilise a two-step approach to offer

For peer review only - http://bmjopen.bmj.com/site/about/guidelines.xhtml

parents analysis for paediatric-onset conditions in their child, adult-onset conditions in themselves, and reproductive carrier screening as a couple three- to six-months after receiving genomic test results for their child.

The challenges of providing multiple types of AF have not been previously investigated in families undergoing testing and the acute care setting raises unique issues. Service models are needed that support the family's autonomy and informed decision-making and are feasible to deliver within a constrained health system. Some key decision points in designing this service included the timing of the offer in relation to the trajectory of the child's illness; design of online ordering and consent forms that account for different family structures and potential privacy concerns; and whether to offer paediatric or adult-onset AF to adolescents and how to support involvement in decision making in this particular group.

Evidence is currently lacking to guide policy and decisions on service delivery models for AF, including uptake; patient experience and preferences; and resource requirements. We will perform a multidisciplinary evaluation to generate comprehensive data across multiple clinical sites, nationally, that will inform future service delivery.

ETHICS AND DISSEMINATION

This project has received ethics approval from the Melbourne Health Human Research Ethics Committee as part of the Australian Genomics Health Alliance protocol: HREC/16/MH/251. All participants will provide written informed consent for data collection, and if relevant, to receive analysis for additional findings. Findings from this study will be

For peer review only

published in peer-reviewed journals and presented at national and international conferences. All participants have provided informed consent to be involved in this study.

AUTHOR CONTRIBUTIONS

Study concept and design was conceived by ZS, CLG, SL. SEB, LL, MM, EL, LD, IG, DFV, SB, BC, JEM, MD, MC and YB contributed to specific evaluation parts of the study and refining study design. IM, LC, GM, EL, LL and SEB were involved in design and delivery of the training for genetic counsellors. LL, FL, DFV, IG, SB and MM will evaluate the data. SEB prepared the first draft of the manuscript. All authors contributed to the drafting of the manuscript, read and approved the final manuscript. In addition to the authors listed, the broader Acute Care Genomics program scientific, clinical, and diagnostic membership had input into this protocol.

FUNDING STATEMENT

This work was supported by the Australian Government's Medical Research Future Fund grant number GHFM76747 and the Royal Children's Hospital Foundation grant number 2020-1259. The authors are funded by Australian Genomics, the Melbourne Genomics Health Alliance, the State Government of Victoria (Department of Health and Human Services) and the Australian Government's Medical Research Future Fund. Australian Genomics is funded by the National Health and Medical Research Council (Grants GNT1113531 and GNT2000001). The research conducted at the Murdoch Children's Research Institute was supported by the Victorian Government's Operational Infrastructure Support Program.

For peer review only

COMPETING INTERESTS

The authors declare no competing interests.

YB and MC are co-Founders of Genetics Adviser, Inc.

REFERENCES

1. Green RC, Berg JS, Grody WW, et al. ACMG recommendations for reporting of incidental findings in clinical exome and genome sequencing. *Genetics in Medicine* 2013;**15**(7):565-74.
2. Tan N, Amendola LM, O'Daniel JM, et al. Is "incidental finding" the best term?: a study of patients' preferences. *Genet Med* 2017;**19**(2):176-81.
3. Bijlsma R, Wouters R, Wessels H, et al. Preferences to receive unsolicited findings of germline genome sequencing in a large population of patients with cancer. *ESMO Open* 2020;**5**(2).
4. Wynn J, Martinez J, Duong J, et al. Research Participants' Preferences for Hypothetical Secondary Results from Genomic Research. *J Genet Couns* 2017;**26**(4):841-51.
5. Vears DF, Minion JT, Roberts SJ, et al. Return of individual research results from genomic research: A systematic review of stakeholder perspectives. *PLoS One* 2021;**16**(11):e0258646.
6. Miller DT, Lee K, Chung WK, et al. ACMG SF v3.0 list for reporting of secondary findings in clinical exome and genome sequencing: a policy statement of the American College of Medical Genetics and Genomics (ACMG). *Genet Med* 2021;**23**(8):1381-90.
7. Downie L, Halliday J, Lewis S, et al. Exome sequencing in newborns with congenital deafness as a modelfor genomic newborn screening: the Baby Beyond Hearing project. *Genetics in Medicine* 2020;**22**(5):937-44.
8. Holm IA, Agrawal PB, Ceyhan-Birsoy O, et al. The BabySeq project: implementing genomic sequencing in newborns. *BMC Pediatr* 2018;**18**(1):225.
9. Van Steijvoort E, Chokoshvili D, J WC, et al. Interest in expanded carrier screening among individuals and couples in the general population: systematic review of the literature. *Hum Reprod Update* 2020;**26**(3):335-55.
10. Jiang S. A Scoping Review of Global Guidelines for the Disclosure of Secondary Genomic Findings to Inform the Establishment of Guidelines in China. *China CDC Wkly* 2022;**4**(32):697-705.
11. Boycott K, Hartley T, Adam S, et al. The clinical application of genome-wide sequencing for monogenic diseases in Canada: Position Statement of the Canadian College of Medical Geneticists. *Journal of Medical Genetics* 2015;**52**(7):431-37.
12. Van El CG, Cornel MC, Borry P, et al. Whole-genome sequencing in health care. *European Journal of Human Genetics* 2013;**21**(6):580-84.
13. Bowdin SC, Hayeems RZ, Monfared N, et al. The SickKids Genome Clinic: developing and evaluating a pediatric model for individualized genomic medicine. *Clin Genet* 2016;**89**(1):10-9.
14. Caulfield M, Davies J, Dennys M, et al. National Genomic Research Library: figshare, 2020.

For peer review only

15. Shahmirzadi L, Chao EC, Palmaer E, et al. Patient decisions for disclosure of secondary findings among the first 200 individuals undergoing clinical diagnostic exome sequencing. *Genet Med* 2014;**16**(5):395-9.
16. Cakici JA, Dimmock DP, Caylor SA, et al. A Prospective Study of Parental Perceptions of Rapid Whole-Genome and -Exome Sequencing among Seriously Ill Infants. *Am J Hum Genet* 2020;**107**(5):953-62.
17. Hill M, Hammond J, Lewis C, et al. Delivering genome sequencing for rapid genetic diagnosis in critically ill children: parent and professional views, experiences and challenges. *Eur J Hum Genet* 2020;**28**(11):1529-40.
18. Smith EE, du Souich C, Dragojlovic N, et al. Genetic counseling considerations with rapid genome-wide sequencing in a neonatal intensive care unit. *J Genet Couns* 2019;**28**(2):263-72.
19. Gyngell C, Lynch F, Stark Z, et al. Consent for rapid genomic sequencing for critically ill children: legal and ethical issues. *Monash Bioeth Rev* 2021;**39**(Suppl 1):117-29.
20. Lynch F, Nisselle A, Stark Z, et al. Genetics follow up after rapid genomic sequencing in intensive care: current practices and recommendations for service delivery. *European Journal of Human Genetics* 2022:1-7.
21. Lynch F, Prentice T, Gillam L, et al. Rapid Genome Sequencing: Consent for New Technologies in the Neonatal Intensive Care Context. *Pediatrics* 2022;**150**(6).
22. Rost C, Dent KM, Botkin J, et al. Experiences and lessons learned by genetic counselors in returning secondary genetic findings to patients. *J Genet Couns* 2020;**29**(6):1234-44.
23. Stavropoulos DJ, Merico D, Jobling R, et al. Whole Genome Sequencing Expands Diagnostic Utility and Improves Clinical Management in Pediatric Medicine. *NPJ Genom Med* 2016;**1**:15012-.
24. Pujol P, Vande Perre P, Faivre L, et al. Guidelines for reporting secondary findings of genome sequencing in cancer genes: the SFMPP recommendations. *Eur J Hum Genet* 2018;**26**(12):1732-42.
25. de Wert G, Dondorp W, Clarke A, et al. Opportunistic genomic screening. Recommendations of the European Society of Human Genetics. *European Journal of Human Genetics* 2021;**29**(3):365-77.
26. Martyn M, Kanga-Parabia A, Lynch E, et al. A novel approach to offering additional genomic findings-A protocol to test a two-step approach in the healthcare system. *J Genet Couns* 2019;**28**(2):388-97.
27. Vu M, Degeling K, Martyn M, et al. Evaluating the resource implications of different service delivery models for offering additional genomic findings. *Genetics in Medicine* 2021;**23**(4):606-13.
28. Schupmann W, Miner SA, Sullivan HK, et al. Exploring the motivations of research participants who chose not to learn medically actionable secondary genetic findings about themselves. *Genet Med* 2021;**23**(12):2281-88.
29. Bombard Y, Hayeems RZ. How digital tools can advance quality and equity in genomic medicine. *Nature Reviews Genetics* 2020;**21**(9):505-06.
30. Shickh S, Rafferty SA, Clausen M, et al. The role of digital tools in the delivery of genomic medicine: enhancing patient-centered care. *Genetics in Medicine* 2021;**23**(6):1086-94.
31. Lunke S, Eggers S, Wilson M, et al. Feasibility of Ultra-Rapid Exome Sequencing in Critically Ill Infants and Children With Suspected Monogenic Conditions in the Australian Public Health Care System. *JAMA* 2020;**323**(24):2503.

For peer review only

32. Harris PA, Taylor R, Minor BL, et al. The REDCap consortium: Building an international community of software platform partners. *J Biomed Inform* 2019;**95**:103208.
33. Harris PA, Taylor R, Thielke R, et al. Research electronic data capture (REDCap)--a metadata-driven methodology and workflow process for providing translational research informatics support. *J Biomed Inform* 2009;**42**(2):377-81.
34. Shickh S, Hirjikaka D, Clausen M, et al. Genetics Adviser: a protocol for a mixed-methods randomised controlled trial evaluating a digital platform for genetics service delivery. *BMJ Open* 2022;**12**(4):e060899.
35. Bombard Y, Clausen M, Shickh S, et al. Effectiveness of the Genomics ADvISER decision aid for the selection of secondary findings from genomic sequencing: a randomized clinical trial. *Genetics in Medicine* 2020;**22**(4):727-35.
36. Bombard Y, Clausen M, Mighton C, et al. The Genomics ADvISER: development and usability testing of a decision aid for the selection of incidental sequencing results. *European Journal of Human Genetics* 2018;**26**(7):984-95.
37. Lawrence CE, Dunkel L, McEver M, et al. A REDCap-based model for electronic consent (eConsent): Moving toward a more personalized consent. *Journal of Clinical and Translational Science* 2020;**4**(4):345-53.
38. Martin AR, Williams E, Foulger RE, et al. PanelApp crowdsources expert knowledge to establish consensus diagnostic gene panels. *Nature Genetics* 2019;**51**(11):1560-65.
39. Stark Z, Foulger RE, Williams E, et al. Scaling national and international improvement in virtual gene panel curation via a collaborative approach to discordance resolution. *The American Journal of Human Genetics* 2021;**108**(9):1551-57.
40. Kirk EP, Ong R, Boggs K, et al. Gene selection for the Australian Reproductive Genetic Carrier Screening Project ("Mackenzie's Mission"). *Eur J Hum Genet* 2021;**29**(1):79-87.
41. Ceyhan-Birsoy O, Machini K, Lebo MS, et al. A curated gene list for reporting results of newborn genomic sequencing. *Genet Med* 2017;**19**(7):809-18.
42. Milko LV, O'Daniel JM, DeCristo DM, et al. An Age-Based Framework for Evaluating Genome-Scale Sequencing Results in Newborn Screening. *J Pediatr* 2019;**209**:68-76.
43. Richards S, Aziz N, Bale S, et al. Standards and guidelines for the interpretation of sequence variants: a joint consensus recommendation of the American College of Medical Genetics and Genomics and the Association for Molecular Pathology. *Genetics in Medicine* 2015;**17**(5):405-23.
44. Marteau TM, Bekker H. The development of a six-item short-form of the state scale of the Spielberger State—Trait Anxiety Inventory (STAI). *British Journal of Clinical Psychology* 1992;**31**(3):301-06.
45. Van Zuuren FJ, De Groot KI, Mulder NL, et al. Coping with medical threat: An evaluation of the Threatening Medical Situations Inventory (TMSI). *Personality and Individual Differences* 1996;**21**(1):21-31.
46. Ong LM, Visser MR, van Zuuren FJ, et al. Cancer patients' coping styles and doctor-patient communication. *Psychooncology* 1999;**8**(2):155-66.
47. Wakefield CE, Homewood J, Mahmut M, et al. Usefulness of the Threatening Medical Situations Inventory in individuals considering genetic testing for cancer risk. *Patient Educ Couns* 2007;**69**(1-3):29-38.
48. O'Connor AM. Validation of a decisional conflict scale. *Med Decis Making* 1995;**15**(1):25-30.

49. Zellerino B, Milligan SA, Brooks R, et al. Development, testing, and validation of a patient satisfaction questionnaire for use in the clinical genetics setting. *Am J Med Genet C Semin Med Genet* 2009;**151c**(3):191-9.
50. Brehaut JC, O'Connor AM, Wood TJ, et al. Validation of a decision regret scale. *Med Decis Making* 2003;**23**(4):281-92.
51. Li M, Bennette CS, Amendola LM, et al. The Feelings About genomiC Testing Results (FACToR) Questionnaire: Development and Preliminary Validation. *J Genet Couns* 2019;**28**(2):477-90.
52. Grant PE, Pampaka M, Payne K, et al. Developing a short-form of the Genetic Counselling Outcome Scale: The Genomics Outcome Scale. *Eur J Med Genet* 2019;**62**(5):324-34.
53. Weiner BJ, Lewis CC, Stanick C, et al. Psychometric assessment of three newly developed implementation outcome measures. *Implementation Science* 2017;**12**(1):108.
54. Proctor E, Silmere H, Raghavan R, et al. Outcomes for implementation research: conceptual distinctions, measurement challenges, and research agenda. *Adm Policy Ment Health* 2011;**38**(2):65-76.

For peer review only

Figure 1: Recruitment flow diagram including survey time points. Microphone denotes timing of appointment recordings. PIS: patient information sheet; GC: genetic counsellor; AF: additional findings

Figure 2: Visual aide for use during pre-test genetic counselling appointment detailing the different types of additional findings

For peer review only

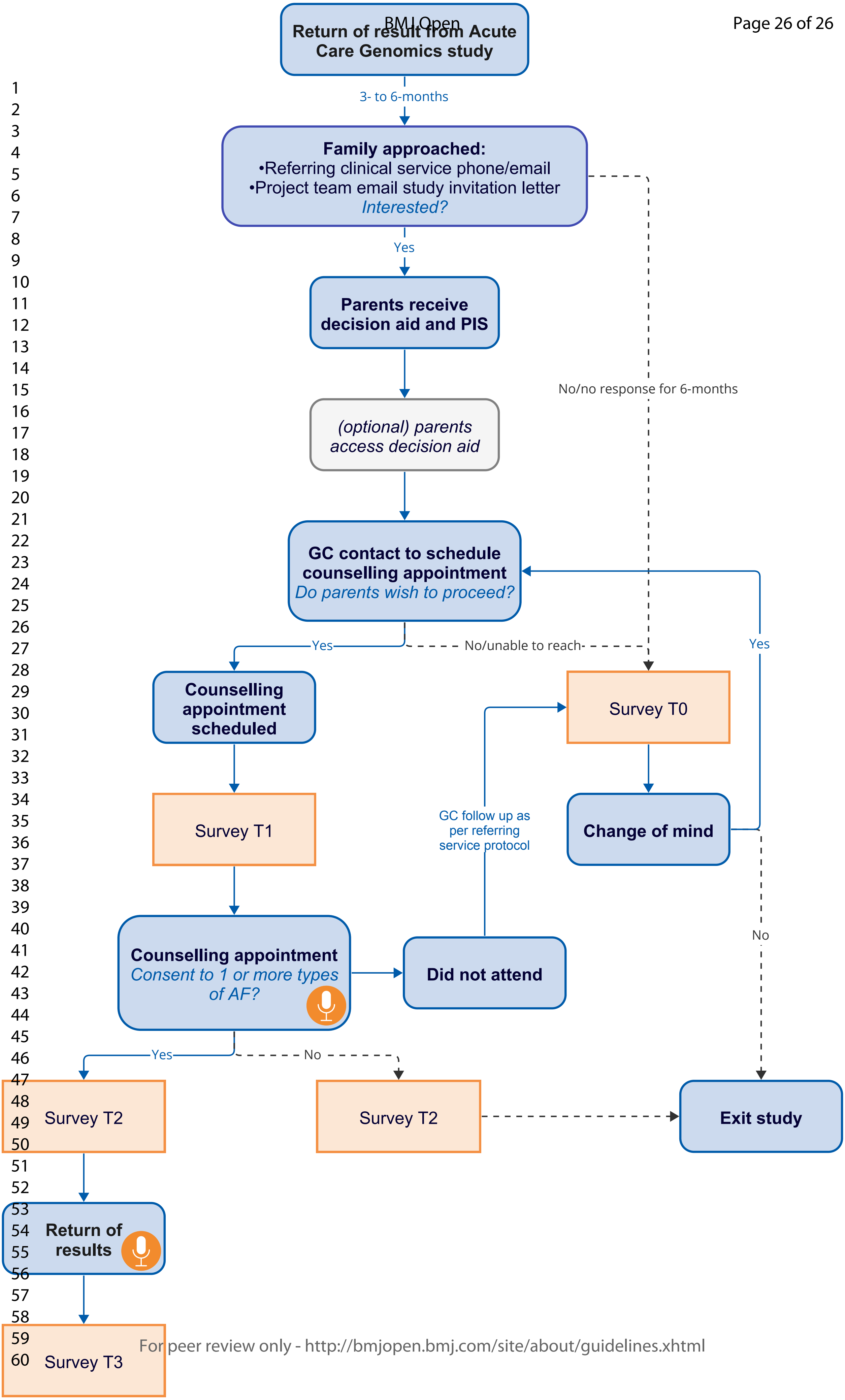
Return of result from Acute Care Genomics study
3- to 6-months
Family approached:
•Referring clinical service phone/email
•Project team email study invitation letter
Interested?
Yes
Parents receive decision aid and PIS
(optional) parents access decision aid
No/no response for 6-months
GC contact to schedule counselling appointment
Do parents wish to proceed?
Yes
No/unable to reach
Yes
Counselling appointment scheduled
Survey T0
Survey T1
GC follow up as per referring service protocol
Change of mind
No
Counselling appointment
Consent to 1 or more types of AF?
Did not attend
Yes
No
Survey T2
Survey T2
Exit study
Return of results
Survey T3

Types of Information Offered

	Childhood-onset Additional Findings	Adult-onset Additional Findings	Genetic Carrier Screening
**Who is the information about?**	Your child	Both parents separately	Both parents together
**What information is being looked for?**	Conditions that affect children. These may or may not have a known treatment or intervention to improve your child's health	Conditions that may develop in adulthood with a known treatment or intervention	Increased chance as a couple of having a child with a different severe or life-threatening genetic condition. Some are treatable, others are not
**Examples**	Muscular dystrophy, some types of blindness or deafness	Hereditary breast and ovarian cancer, hereditary bowel cancer, genetic heart conditions	Carrier for cystic fibrosis, sickle cell disease, some forms of developmental delay
**Impact**	Can reveal your child's chance of developing another genetic condition	You may need more tests and procedures	Can impact pregnancy planning

For peer review only - http://bmjopen.bmj.com/site/about/guidelines.xhtml

BMJ Open

A study protocol for the two-step offer and return of multiple types of additional genomic findings to families after ultra-rapid trio genomic testing in the acute care setting

Journal:	*BMJ Open*
Manuscript ID	bmjopen-2023-072999.R1
Article Type:	Protocol
Date Submitted by the Author:	08-May-2023
Complete List of Authors:	Bouffler, Sophie; Australian Genomics Health Alliance,<br>Lee, Ling; Walter and Eliza Hall Institute of Medical Research, Melbourne Genomics Health Alliance ; Murdoch Children's Research Institute<br>Lynch, Fiona; Murdoch Children's Research Institute; The University of Melbourne<br>Martyn, Melissa; Walter and Eliza Hall Institute of Medical Research, Melbourne Genomics Health Alliance ; Murdoch Children's Research Institute<br>Lynch, Elly; Walter and Eliza Hall Institute of Medical Research, Melbourne Genomics Health Alliance ; Victorian Clinical Genetics Services Ltd<br>Macciocca, Ivan; The University of Melbourne; Victorian Clinical Genetics Services Ltd<br>Curnow, Lisette; Victorian Clinical Genetics Services Ltd<br>McCorkell, Giulia; Australian Genomics Health Alliance; The University of Melbourne<br>Lunke, Sebastian; Victorian Clinical Genetics Services Ltd; The University of Melbourne<br>Chong, Belinda; Victorian Clinical Genetics Services Ltd<br>Marum, Justine E; Victorian Clinical Genetics Services Ltd<br>Delatycki, Martin; Victorian Clinical Genetics Services Ltd; The University of Melbourne<br>Downie, Lilian; Murdoch Children's Research Institute; The University of Melbourne<br>Goranitis, I; The University of Melbourne; Australian Genomics Health Alliance<br>Vears, Danya F; Murdoch Children's Research Institute; The University of Melbourne<br>Best, Stephanie; Australian Genomics Health Alliance; Peter MacCallum Cancer Centre<br>Clausen, Marc; Unity Health Toronto, Genomics Health Services Research Program, St. Michael's Hospital<br>Bombard, Yvonne; Unity Health Toronto, Genomics Health Services Research Program, St. Michael's Hospital; University of Toronto Institute of Health Policy Management and Evaluation<br>Stark, Zornitza; Australian Genomics Health Alliance; Victorian Clinical Genetics Services Ltd<br>Gaff , Clara; Walter and Eliza Hall Institute of Medical Research,

	Melbourne Genomics Health Alliance ; The University of Melbourne
**Primary Subject Heading**:	Genetics and genomics
Secondary Subject Heading:	Health services research, Qualitative research
Keywords:	GENETICS, Paediatric intensive & critical care < PAEDIATRICS, Protocols & guidelines < HEALTH SERVICES ADMINISTRATION & MANAGEMENT, Neonatal intensive & critical care < INTENSIVE & CRITICAL CARE, PAEDIATRICS, QUALITATIVE RESEARCH

SCHOLARONE™ Manuscripts

A study protocol for the two-step offer and return of multiple types of additional genomic findings to families after ultra-rapid trio genomic testing in the acute care setting

Sophie E Bouffler[1], Ling Lee[2,3], Fiona Lynch[3,4], Melissa Martyn[2,3], Elly Lynch[2,5], Ivan Macciocca[4,5], Lisette Curnow[5], Giulia McCorkell[1,4], Sebastian Lunke[4,5], Belinda Chong[5], Justine E Marum[5], Martin Delatycki[4,5], Lilian Downie[3,5], Ilias Goranitis[1,6], Danya F Vears[3,4], Stephanie Best[1,7], Marc Clausen[8], Yvonne Bombard[8,9], Zornitza Stark[1,5], Clara L Gaff[2,3]

[1]Australian Genomics, Melbourne, Australia

[2]Melbourne Genomics Health Alliance, Melbourne, VIC, Australia

[3]Murdoch Children's Research Institute, Melbourne, VIC, Australia

[4]University of Melbourne, Melbourne, Australia

[5]Victorian Clinical Genetics Services, Murdoch Children's Research Institute, Melbourne, Australia

[6]Health Economics Unit, Centre for Health Policy, Melbourne School of Population and Global Health, University of Melbourne, Melbourne, Australia

[7]Peter MacCallum Cancer Centre, Melbourne, Australia

[8] Genomics Health Services Research Program, St. Michael's Hospital, Unity Health Toronto, Toronto, ON, Canada

[9]Institute of Health Policy, Management and Evaluation, University of Toronto, Toronto, ON, Canada

For peer review only

Corresponding author: Clara Gaff, clara.gaff@melbournegenomics.org.au, Melbourne Genomics Health Alliance, Melbourne, VIC, Australia, Murdoch Children's Research Institute, Melbourne, VIC, Australia

Word count: 3440

Keywords: Additional findings, genomics, acute care, decision support

For peer review only

ABSTRACT

Introduction As routine genomic testing expands, so too does the opportunity to look for additional health information unrelated to the original reason for testing, termed additional findings (AF). Analysis for many different types of AF may be available, particularly to families undergoing trio genomic testing. The optimal model for service delivery remains to be determined, especially when the original test occurs in the acute care setting.

Methods and analysis Families enrolled in a national study providing ultra-rapid genomic testing to critically ill children will be offered analysis for three types of AF on their stored genomic data: paediatric-onset conditions in the child, adult-onset conditions in each parent, and reproductive carrier screening for the parents as a couple. The offer will be made three- to six-months after diagnostic testing. Parents will have access to a modified version of the Genetics Adviser web-based decision support tool before attending a genetic counselling appointment to discuss consent for AF. Parental experiences will be evaluated using qualitative and quantitative methods on data collected through surveys, appointment recordings, and interviews at multiple timepoints. Evaluation will focus on parental preferences, uptake, decision support use, and understanding of AF. Genetic health

professionals' perspectives on acceptability and feasibility of AF will also be captured through surveys and interviews.

Ethics and dissemination This project received ethics approval from the Melbourne Health Human Research Ethics Committee as part of the Australian Genomics Health Alliance protocol: HREC/16/MH/251. Findings will be disseminated through peer-review journal articles and at conferences nationally and internationally.

For peer review only

ARTICLE SUMMARY

Strengths and limitations of this study

- This is a prospective national pilot study across 17 clinical sites exploring the offer and receipt of three types of AF following genomic testing in the acute care setting.
- This study will investigate the practicability, acceptability, and value of offering AF analysis to parents and children using stored genomic data 3-6 months after diagnostic testing.
- Results from this study can inform future health system implementation of analysis for AF.
- A process evaluation will be undertaken to investigate parental decision-making and preferences for health service delivery.
- This study explores AF specifically in the context of acute neonatal and paediatric care.

INTRODUCTION

Additional findings (AF) refer to results from genomic testing that may have medical value and/or utility and are found in a deliberate search unrelated to the original reason for

testing.[1,2] Such findings may include adult- and paediatric-onset treatable and untreatable disorders, reproductive carrier status, and pharmacogenomic testing. Research participants consistently express a high level of interest in receiving AF.[3-5]

The American College of Medical Genetics and Genomics (ACMG) initially advocated the return of adult-onset AF as a mandatory part of genomic testing regardless of the patient's age,[1] with updated guidelines suggesting an opt-in approach with a minimum list of 73 genes to be reported.[6] The offer of paediatric-onset AF for children undergoing genomic sequencing has been explored, often as a prelude to developing newborn screening programs.[7,8] Stored genomic data can also be utilised for reproductive genetic carrier screening. As carrier screening becomes more common[9] it is a natural extension for parents undergoing trio genomic testing to be offered this additional information as they are typically of child-bearing age. A recent systematic review[10] summarised the position of several countries, with many international bodies, including the Canadian College of Medical Geneticists and the European Society of Human Genetics (ESHG), supporting a more conservative approach and recommended that analysis and reporting from genomic sequencing should be focused on the primary reason for testing.[11,12] In Australia, analysis is largely restricted to genes known to be associated with the patient's primary clinical indication for testing.

Determining whether and how to deliver AF within health services continues to be debated by professional bodies. International studies have described models whereby the offer of AF is made at the time diagnostic testing is initiated.[13-15] There are concerns that this model adds to the complexity of pre-test counselling at a time when families are already

For peer review only

overwhelmed by having to make many complex decisions. This is particularly pertinent in the acute neonatal and paediatric care setting, when parents are dealing with decision-making for their critically ill child in a highly stressful, time pressured environment. Concerns have been raised about parents' ability to process and retain information and the impact of genomic results on family functioning in this setting.[16-21] Emerging evidence supports the validity of these concerns,[22] and in one study the offer of AF deterred families from receiving genetic testing when a diagnostic result would be in the child's best interest.[23] The ESHG have more recently acknowledged the method proposed by the French Society of Predictive and Personalized Medicine, which recommends a multi-step approach to consent for AF,[24] but note the need for further empirical evidence.[25]

The Melbourne Genomics Health Alliance described a pilot study trialling a two-step offer for AF analysis on stored data in adult patients, with the offer occurring subsequent to the primary result return.[26] This model allows for temporal separation of the offer and avoids burdening patients with complex decisions at a time of high stress, while still offering them the opportunity to receive more health information from their stored genomic data.

The routine provision of AF in a two-step model could involve a significant investment of time and resources.[27] As evidence reinforcing the importance of providing detailed information during the informed consent process in this space emerges[28] it is essential to explore tools that will support this. Digital decision support tools have the potential to alleviate some of the resource constraints when implemented in a genomic care pathway, alongside in-person encounters.[29,30]

For peer review only

As genomic sequencing is increasingly incorporated into different clinical settings there continues to be a need for evidence to inform decisions about how healthcare systems should manage AF. Storage of genomic data enables analysis for AF as a separate decision after diagnostic testing is complete.

The Acute Care Genomics (ACG) study[31] is evaluating ultra-rapid trio whole genome sequencing for critically ill infants and children on a national scale. The study cohort includes parents and children who have stored whole genome sequencing data from trio testing. This cohort provides the opportunity to understand the service implications of offering and returning multiple AF in acute care on a national scale, as well as parental preferences for receiving this information, across multiple genetic services.

Study aim

We describe a prospective national study offering analysis for multiple types of additional findings on stored data to children and their parents undergoing trio genomic testing via a two-step delivery model. This study uses process evaluation to examine decision-making, decision support, counselling processes, as well as resource and service implications.

METHODS AND ANALYSIS

Eligibility and recruitment

This study will recruit families via a two-step model as described in a previous study.[26] The ACG study will offer up to 250 families ultra-rapid genomic trio/duo testing at 17 clinical sites around Australia between July 2020 and April 2022. As part of their participation in the ACG study, parents will be approached three- to six-months after disclosure of the child's

For peer review only

ultra-rapid genomic testing result to be offered analysis for AF using their stored genomic data. Research has shown that parents commonly try to conceive shortly after experiencing a perinatal loss.[32,33] To best support families in this situation, parents who enquire seeking recurrence or reproductive risk information before the study-prompted offer of AF may have the opportunity for earlier analysis. This study will run from July 2020 to June 2023. Study data will be managed using REDCap electronic data capture tools hosted at the Murdoch Children's Research Institute, Melbourne, Australia.[34,35]

Families will be excluded where the child is still a hospital inpatient, or if the proband's death is under coroner's investigation. Under exceptional circumstances, if the referring clinical team deem it inappropriate to approach a family for psychosocial reasons they will be excluded. Examples of such circumstances may be families experiencing high levels of psychological distress, cases where the parents are in the process of relinquishing care of the child, or if one parent is no longer involved in the child's care.

Families will be eligible to receive three types of AF:

1. Paediatric-onset additional findings in the child
2. Adult-onset additional findings in each parent
3. Expanded couple genetic carrier screening

Where the child is deceased, paediatric-onset AF will not be offered but testing will be offered to the parents. Expanded couple genetic carrier screening will not be offered for duos (one parent and child), or where the parents are separated.

For peer review only

Study design

The study design is outlined in Figure 1.

The study has been designed to minimise distress to parents wherever possible, noting the increased sensitivity around parents in the acute care setting. To reflect expected real-world service delivery and facilitate continuity of care, families will be approached by the original referring clinical service and the same provider who saw them for the initial testing where feasible. Alternatively, the study team will send an approach letter via email including a link to a survey asking parents for their expression of interest. Parents will be approached in separate emails if the referring team so advise.

Parents who express interest will receive login details for a web-based decision support tool and a copy of the patient information sheet. The counselling team will subsequently contact families to arrange a pre-test genetic counselling appointment to discuss consent. All families must meet with a genetic health professional to proceed with receiving AF.

Decision support

The study will deploy an interactive web-based decision support tool, called Genetics Adviser,[36] as an adjunct to a genetic counselling appointment. The tool is an expanded version of the Genomics ADvISER, (a genomics decision AiD about Incidental SEquencing Results), which has been shown to effectively augment genetic counselling and improve patient knowledge and decisional confidence.[30,37] The Genetics Adviser is a comprehensive, interactive, patient-centred platform aimed at providing lay language digital content to support informed decision making, offer pre- and post- genetic testing support, and

For peer review only

facilitate return of results. The tool can be tailored for particular use cases as well as a range of clinical and population testing scenarios.[36] For this protocol, the Genetics Adviser was customised to focus on pre-test counselling and education for AF with the support of genetic counselling in this process.[38] Content has been adapted in collaboration with the Genetics Adviser team to enable the delivery of tailored information on the three types of AF offered in this study with a focus on using plain language for the content and videos. To minimise distress to parents of children who have died, an alternative version of the tool will be used for these parents, which omits the section on paediatric-onset additional findings.

Each parent will receive separate login details for the Genetics Adviser and will be encouraged to work through the tool individually prior to the pre-test genetic counselling appointment.

Genetic counselling and consent

All families will receive genetic counselling. Pre- and post-test genetic counselling appointments will be conducted in person or via telehealth. Families will be asked for consent to audio record pre- and post-test genetic counselling appointments following an approved verbal consent script.

Genetic health professionals who will conduct pre-test counselling for AF will be invited to an online education workshop facilitated by genetic counsellors with experience delivering AF in a research setting. The workshop will include lessons from previous studies offering AF, small group role plays of AF counselling scenarios, and detailed study logistics. There will

For peer review only

be a pre-workshop self-directed module including two video recorded simulation pre-test counselling appointments using trained counsellors and actors. The participating genetic health professionals will be provided with talking points and tips for counselling families to ensure that the information given and language used is consistent between services, as well as a visual aide summarising the different types of AF (Figure 2).

All participants will provide informed consent for data collection as part of their involvement in the Acute Care Genomics study.[31] This consent encompasses the research component of the AF study, with parents providing separate clinical consent to the different types of AF.

Clinical consent for AF will be captured via the REDCap e-consent framework.[39] For the majority of families, consent will be documented using a single clinical e-consent form that displays all of the AF offerings the family are eligible for. Where the referring team indicate the decision for adult-onset AF will be made in separate appointments, the genetic health professional will have access to individual e-consent forms for each parent. Consent from one parent is required to proceed with analysis for paediatric-onset AF in the child; however, the genetic health professional will discuss this decision with both parents and ensure there is a consensus where possible as per standard clinical practice. Both parents must provide consent for couple genetic carrier screening to proceed.

All results will be returned by a genetic health professional in person or via telehealth/phone. All results will be returned simultaneously, unless the couple are

For peer review only

pregnant and have requested carrier testing, in which case the results may be returned early to facilitate reproductive planning.

Gene lists

All gene lists have been developed in previous studies, using structured frameworks and expert groups to define content. The gene list for the Australian Reproductive Carrier Screening Project (Mackenzie's Mission_Reproductive Carrier Screening) will be used for couple carrier screening.[40] Paediatric-onset additional findings (Additional findings_Paediatric) will encompass genes tested as part of the BabySeq project,[41] NC NEXUS study,[42] and Baby Beyond Hearing project.[7] The final list includes treatable and untreatable conditions affecting children. The gene list for adult-onset additional findings (Additional findings_Adult) is based on the list created for the Melbourne Genomics Additional Findings flagship,[26] which includes genes associated with clinically actionable conditions where a publicly funded management pathway exists. This list was updated to include genes on the ACMG V3.0 Secondary Findings list.[6]

Gene lists to guide analysis for the different types of AF are available on PanelApp Australia (https://panelapp.agha.umccr.org/).[43,44] The content on this platform is openly available.

Laboratory protocol

The laboratory will receive separate requests for each type of AF to which a family has given consent. The laboratory will analyse the stored genomic data according to National Association of Testing Authorities (NATA) clinically accredited procedures. Variants will be

For peer review only

classified based on ACMG classification guidelines[45] and only pathogenic or likely pathogenic variants will be reported. All variants identified for reporting will be discussed in a multidisciplinary team meeting, including clinical geneticists, other relevant medical subspecialists, genetic counsellors, bioinformaticians, and molecular geneticists. Variants of uncertain significance will not be reported, in line with population screening guidelines.[46] The standard turnaround time for AF reports will be 12 weeks. Reproductive carrier screening results will be expedited if clinically indicated.

Where the child has previously been diagnosed with an autosomal recessive, X-linked or inherited dominant condition, increased reproductive risk will be included on the couple carrier screening report, regardless of whether the gene is contained in the carrier screening panel.

Evaluation

This study will answer the following questions:

1. What is the uptake of the different types of AF?
2. What influences people's decisions to accept or decline the different types of AF?
3. How is the decision support tool used?
4. What are stakeholder perspectives of the decision support tool?
5. To what extent do counselling tools and processes support understanding of AF?
6. What are stakeholders' (patients and genetic health professionals) views of offering AF in the acute care setting?

Clinical and genetic counselling data collection

For peer review only

We will collect data on how many families are offered, accept, and decline AF analysis and at which stage of the process this occurs. We will collect data on the types of AF families choose to have and the outcomes of analysis. Data will also be collected on laboratory turnaround times. Pre- and post-test genetic counselling appointments will be audio recorded.

Surveys: Parents

Participants will be invited to prospectively complete surveys throughout the study (see Figure 1). All surveys will be delivered electronically to each parent via the study's REDCap database. Participants will be invited to complete the first survey (T1) prior to the pre-test counselling appointment, the second (T2) after deciding whether to accept any or decline all AF and the third (T3) following result return. Survey responses will be linked between timepoints. If participants decline before counselling, they will receive a decliner survey (T0); immediately for active decliners (parents who explicitly decline AF) or six months after approach for passive decliners (parents who do not respond to contact from the clinical or study team).

Survey measures are outlined in

Table 1. T1 will capture perspectives on the decision tool, parent understanding of the AF offer, and baseline psychometrics. T2 will evaluate processes and reasons for decision-making as well as parental values and understanding of analysis for AF post-counselling. T3 will capture information on the participant's values, experience and understanding of AF results.

For peer review only

Table 1: Survey measures

Measure	Description	T1	T2	T3	T0
Decision support tool user assessment	Six study-specific questions about decision support use, clarity, relevance, bias, other information needs and areas of improvement.	X			
Knowledge of AF	Seven study-specific questions to determine a participants' understanding of AF offered.	X	X		
State trait anxiety index (STAI-AD)	26-item scale measuring state and trait anxiety.<br>*Copyright © 1968, 1977 by Charles D. Spielberger. All rights reserved in all media. Published by Mind Garden,Inc.* www.mindgarden.com<br>Note: The 6 questions that form the STAI-6 short form[47] are repeated at each timepoint. If participants complete any of the remaining 20 items in any survey they will not be asked these items again.	X	X	X	X
Health behaviour	A shortened version of the Threatening Medical Situations Inventory.[48-50]	X	X		X
Demographics	Age, gender, education, income, language, marital status, number of children, prior experience with genomic testing, family planning, private health insurance status, postcode.<br>Note: if participants complete demographic questions in any survey they will not be asked again.	X	X	X	X
Decision recall	One study-specific question to assess whether parents recall their decision.		X	X	
Difficulty and deliberation of decision	Two study-specific questions to assess how difficult it was and how long it took parents to decide which AF to receive (T2) or to decline (T0)		X		X
Reasons for accepting or declining	Three study-specific questions for each type of AF offered addressing reasons for the participant's decision. Participants are asked to rate a selection of reasons on a 5-point Likert scale and asked to comment if there are other reasons not listed.<br>Note: separate questions for acceptors and decliners of the different types of AF		X		
Decisional conflict scale	16-item scale measuring decisional conflict.[51]		X		

For peer review only

Acceptable information needs	Two study-specific questions about whether use of the decision tool alone without genetic counselling would provide enough information to make a decision.		X		
Genetic counselling satisfaction	Seven-item scale addressing patient satisfaction and service quality in the clinical genetics setting.[52]		X	X	
Willingness to pay	Dynamic triple-bounded dichotomous choice contingent valuation, also known as a 'bidding game' to assess the value participants place on the information that comes from analysis for AF.		X	X	
Results recall and understanding of results	Four study-specific questions to determine if participants recalled their results correctly, and if they understand what these results mean.			X	
Future planning	Five study-specific questions on future planning for themselves, their child, and their family based on the AF results received.			X	
Decision regret scale	Five-item scale to measure regret after a health care decision.[53]			X	
FACToR questionnaire	12-item questionnaire adapted to assess the psychosocial impact of returning genomic test results.[54]			X	
Genomics outcome scale (GOS)	Six-item scale based on the Genetic Counselling Outcome Scale (GCOS-24) adapted to assess outcomes of genetic counselling and patient empowerment.[55]			X	
Service delivery preferences	Three study-specific questions addressing how and when the offer for AF should be made.			X	
Value of offering AF	One study-specific question on the value of information that comes from analysis for AF. Participants are asked to rate different factors on a 4-point Likert scale from extremely valuable to not valuable.			X	
Reasons for declining	One study-specific question exploring the reasons for declining. Participants are presented with 13 reasons and asked to rate how much each one influenced their decision on a scale of 1 (did not influence) to 5 (strongly influenced).				X

AF – additional findings, T1 – pre-test counselling survey, T2 – post-decision for AF survey, T3 – post-result return survey, T0 – passive or active decliner survey

For peer review only

Parent interviews

Participants will be invited to provide their contact details during any of the surveys if they are willing to be contacted for an interview to discuss their experience of the study in greater detail. Those who do so will be contacted a minimum of three months after result return or after they decline AF and offered the option to participate in a semi-structured interview, either by phone or via an online platform (see Figure 1). The interviews will explore participants' experiences with the web-based decision support tool, genetic counselling appointment, consent process, and result disclosure. Interviews will be audio-recorded, transcribed, deidentified, and analysed using inductive content analysis.

Genetic health professional evaluation

The perspectives of genetic health professionals (genetic counsellors and clinical geneticists) implementing AF in this study will be evaluated using a mixed methods study. Participating health professionals will be identified by the study team and receive a validated survey[56] prior to commencing the AF study with families and upon completion of result return to the final participants. The survey aims to establish practitioners' perspectives of the implementation outcomes of acceptability, appropriateness, and feasibility.[57] Follow up semi-structured interviews will be undertaken through an online platform to investigate influences on clinicians' perspectives and to identify enablers to the adoption of AF for practitioners. Transcripts will be audio recorded and transcribed before deductive and inductive data analysis is undertaken.

Data analysis

For peer review only - http://bmjopen.bmj.com/site/about/guidelines.xhtml

Overall AF uptake will be reported as the number of families who accept any type of AF divided by the total offered any AF. Uptake of each type of AF will also be reported, noting that the denominator for each AF offer may differ.

Quantitative data from surveys (Likert-scales, categorical items) will be analysed using standard statistical methodology. If sample sizes for each group provide sufficient power, multivariate statistical analyses will be performed to explore factors that may influence uptake (e.g., received primary diagnosis or not, family completion status). Economic implications associated with the delivery of AF and the effect on follow-on care pathways will be evaluated using health economic modelling methods, subject to group size. Quantitative data on decision support tool uptake and utilisation will be analysed. Uptake will be measured by determining the number of parents who accessed the Genetics Adviser divided by the number of parents who were invited. Utilisation will be measured by the time parents took to work through the Genetics Adviser and the drop off rate.

Qualitative data will include open-text survey responses and audio recordings (of interviews and genetic counselling sessions). Open-text survey responses will be analysed iteratively, using inductive content analysis, and used to explain the quantitative survey findings. The approach to analysis of counselling transcripts will be informed by responses to surveys and decision support tool utilisation metrics. It is anticipated that a hybrid deductive-inductive content analysis approach will be used to code counselling interactions to explore the impact of use of the decision support tool on counselling and decision making and identify counselling challenges (specific to the acute care setting).

For peer review only

Patient and Public Involvement

The design and delivery of this study has been heavily informed by previous consumer focused studies,[7,26] incorporating learnings from participant responses and feedback gathered in these studies. The study also uses Genetics Adviser, a decision support platform with extensive public input into design and development.[30,36-38]

DISCUSSION

This protocol describes the offer and return of three categories of AF to families participating in a study examining national implementation of ultra-rapid genomic diagnostic testing in the acute care setting. We will utilise a two-step approach to offer parents analysis for paediatric-onset conditions in their child, adult-onset conditions in themselves, and reproductive carrier screening as a couple three- to six-months after receiving genomic test results for their child.

The challenges of providing multiple types of AF have not been previously investigated in families undergoing testing and the acute care setting raises unique issues. Service models are needed that support the family's autonomy and informed decision-making and are feasible to deliver within a constrained health system. Some key decision points in designing this service included the timing of the offer in relation to the trajectory of the child's illness; design of online ordering and consent forms that account for different family structures and potential privacy concerns; and whether to offer paediatric or adult-onset AF to adolescents and how to support involvement in decision making in this particular group.

For peer review only

The sample size for this protocol is pre-determined by recruitment into the larger Acute Care Genomics study. This will be considered when reporting the findings of our study and we acknowledge that there may still be a need for larger, purposefully designed studies in the future.

Evidence is currently lacking to guide policy and decisions on service delivery models for AF, including uptake; patient experience and preferences; and resource requirements. We will perform a multidisciplinary evaluation to generate comprehensive data across multiple clinical sites, nationally, that will inform future service delivery.

ETHICS AND DISSEMINATION

This project has received ethics approval from the Melbourne Health Human Research Ethics Committee as part of the Australian Genomics Health Alliance protocol: HREC/16/MH/251. All participants will provide written informed consent for data collection, and if relevant, to receive analysis for additional findings. Findings from this study will be published in peer-reviewed journals and presented at national and international conferences. All participants have provided informed consent to be involved in this study.

AUTHOR CONTRIBUTIONS

Study concept and design was conceived by ZS, CLG, SL. SEB, LL, MM, EL, LD, IG, DFV, SB, BC, JEM, MD, MC and YB contributed to specific evaluation parts of the study and refining study design. IM, LC, GM, EL, LL and SEB were involved in design and delivery of the training for genetic counsellors. LL, FL, DFV, IG, SB and MM will evaluate the data. SEB prepared the first draft of the manuscript. All authors contributed to the drafting of the manuscript, read and

For peer review only

approved the final manuscript. In addition to the authors listed, the broader Acute Care Genomics program scientific, clinical, and diagnostic membership had input into this protocol.

FUNDING STATEMENT

This work was supported by the Australian Government's Medical Research Future Fund grant number GHFM76747 and the Royal Children's Hospital Foundation grant number 2020-1259. The authors are funded by Australian Genomics, the Melbourne Genomics Health Alliance (grant number N/A), the State Government of Victoria (Department of Health and Human Services; grant number N/A), and the Australian Government's Medical Research Future Fund. Australian Genomics is funded by the National Health and Medical Research Council (Grants GNT1113531 and GNT2000001). The research conducted at the Murdoch Children's Research Institute was supported by the Victorian Government's Operational Infrastructure Support Program.

COMPETING INTERESTS

The authors declare no competing interests.

YB and MC are co-Founders of Genetics Adviser, Inc.

REFERENCES

1. Green RC, Berg JS, Grody WW, et al. ACMG recommendations for reporting of incidental findings in clinical exome and genome sequencing. *Genetics in Medicine* 2013;**15**(7):565-74.
2. Tan N, Amendola LM, O'Daniel JM, et al. Is "incidental finding" the best term?: a study of patients' preferences. *Genet Med* 2017;**19**(2):176-81.

3. Bijlsma R, Wouters R, Wessels H, et al. Preferences to receive unsolicited findings of germline genome sequencing in a large population of patients with cancer. *ESMO Open* 2020;**5**(2).
4. Wynn J, Martinez J, Duong J, et al. Research Participants' Preferences for Hypothetical Secondary Results from Genomic Research. *J Genet Couns* 2017;**26**(4):841-51.
5. Vears DF, Minion JT, Roberts SJ, et al. Return of individual research results from genomic research: A systematic review of stakeholder perspectives. *PLoS One* 2021;**16**(11):e0258646.
6. Miller DT, Lee K, Chung WK, et al. ACMG SF v3.0 list for reporting of secondary findings in clinical exome and genome sequencing: a policy statement of the American College of Medical Genetics and Genomics (ACMG). *Genet Med* 2021;**23**(8):1381-90.
7. Downie L, Halliday J, Lewis S, et al. Exome sequencing in newborns with congenital deafness as a modelfor genomic newborn screening: the Baby Beyond Hearing project. *Genetics in Medicine* 2020;**22**(5):937-44.
8. Holm IA, Agrawal PB, Ceyhan-Birsoy O, et al. The BabySeq project: implementing genomic sequencing in newborns. *BMC Pediatr* 2018;**18**(1):225.
9. Van Steijvoort E, Chokoshvili D, J WC, et al. Interest in expanded carrier screening among individuals and couples in the general population: systematic review of the literature. *Hum Reprod Update* 2020;**26**(3):335-55.
10. Jiang S. A Scoping Review of Global Guidelines for the Disclosure of Secondary Genomic Findings to Inform the Establishment of Guidelines in China. *China CDC Wkly* 2022;**4**(32):697-705.
11. Boycott K, Hartley T, Adam S, et al. The clinical application of genome-wide sequencing for monogenic diseases in Canada: Position Statement of the Canadian College of Medical Geneticists. *Journal of Medical Genetics* 2015;**52**(7):431-37.
12. Van El CG, Cornel MC, Borry P, et al. Whole-genome sequencing in health care. *European Journal of Human Genetics* 2013;**21**(6):580-84.
13. Bowdin SC, Hayeems RZ, Monfared N, et al. The SickKids Genome Clinic: developing and evaluating a pediatric model for individualized genomic medicine. *Clin Genet* 2016;**89**(1):10-9.
14. Caulfield M, Davies J, Dennys M, et al. National Genomic Research Library: figshare, 2020.
15. Shahmirzadi L, Chao EC, Palmaer E, et al. Patient decisions for disclosure of secondary findings among the first 200 individuals undergoing clinical diagnostic exome sequencing. *Genet Med* 2014;**16**(5):395-9.
16. Cakici JA, Dimmock DP, Caylor SA, et al. A Prospective Study of Parental Perceptions of Rapid Whole-Genome and -Exome Sequencing among Seriously Ill Infants. *Am J Hum Genet* 2020;**107**(5):953-62.
17. Hill M, Hammond J, Lewis C, et al. Delivering genome sequencing for rapid genetic diagnosis in critically ill children: parent and professional views, experiences and challenges. *Eur J Hum Genet* 2020;**28**(11):1529-40.
18. Smith EE, du Souich C, Dragojlovic N, et al. Genetic counseling considerations with rapid genome-wide sequencing in a neonatal intensive care unit. *J Genet Couns* 2019;**28**(2):263-72.
19. Gyngell C, Lynch F, Stark Z, et al. Consent for rapid genomic sequencing for critically ill children: legal and ethical issues. *Monash Bioeth Rev* 2021;**39**(Suppl 1):117-29.

For peer review only

20. Lynch F, Nisselle A, Stark Z, et al. Genetics follow up after rapid genomic sequencing in intensive care: current practices and recommendations for service delivery. *European Journal of Human Genetics* 2022:1-7.
21. Lynch F, Prentice T, Gillam L, et al. Rapid Genome Sequencing: Consent for New Technologies in the Neonatal Intensive Care Context. *Pediatrics* 2022;**150**(6).
22. Rost C, Dent KM, Botkin J, et al. Experiences and lessons learned by genetic counselors in returning secondary genetic findings to patients. *J Genet Couns* 2020;**29**(6):1234-44.
23. Stavropoulos DJ, Merico D, Jobling R, et al. Whole Genome Sequencing Expands Diagnostic Utility and Improves Clinical Management in Pediatric Medicine. *NPJ Genom Med* 2016;**1**:15012-.
24. Pujol P, Vande Perre P, Faivre L, et al. Guidelines for reporting secondary findings of genome sequencing in cancer genes: the SFMPP recommendations. *Eur J Hum Genet* 2018;**26**(12):1732-42.
25. de Wert G, Dondorp W, Clarke A, et al. Opportunistic genomic screening. Recommendations of the European Society of Human Genetics. *European Journal of Human Genetics* 2021;**29**(3):365-77.
26. Martyn M, Kanga-Parabia A, Lynch E, et al. A novel approach to offering additional genomic findings-A protocol to test a two-step approach in the healthcare system. *J Genet Couns* 2019;**28**(2):388-97.
27. Vu M, Degeling K, Martyn M, et al. Evaluating the resource implications of different service delivery models for offering additional genomic findings. *Genetics in Medicine* 2021;**23**(4):606-13.
28. Schupmann W, Miner SA, Sullivan HK, et al. Exploring the motivations of research participants who chose not to learn medically actionable secondary genetic findings about themselves. *Genet Med* 2021;**23**(12):2281-88.
29. Bombard Y, Hayeems RZ. How digital tools can advance quality and equity in genomic medicine. *Nature Reviews Genetics* 2020;**21**(9):505-06.
30. Shickh S, Rafferty SA, Clausen M, et al. The role of digital tools in the delivery of genomic medicine: enhancing patient-centered care. *Genetics in Medicine* 2021;**23**(6):1086-94.
31. Lunke S, Eggers S, Wilson M, et al. Feasibility of Ultra-Rapid Exome Sequencing in Critically Ill Infants and Children With Suspected Monogenic Conditions in the Australian Public Health Care System. *JAMA* 2020;**323**(24):2503.
32. Lamb EH. The impact of previous perinatal loss on subsequent pregnancy and parenting. *J Perinat Educ* 2002;**11**(2):33-40.
33. Skjaerven R, Wilcox AJ, Lie RT, et al. Selective fertility and the distortion of perinatal mortality. *Am J Epidemiol* 1988;**128**(6):1352-63.
34. Harris PA, Taylor R, Minor BL, et al. The REDCap consortium: Building an international community of software platform partners. *J Biomed Inform* 2019;**95**:103208.
35. Harris PA, Taylor R, Thielke R, et al. Research electronic data capture (REDCap)--a metadata-driven methodology and workflow process for providing translational research informatics support. *J Biomed Inform* 2009;**42**(2):377-81.
36. Shickh S, Hirjikaka D, Clausen M, et al. Genetics Adviser: a protocol for a mixed-methods randomised controlled trial evaluating a digital platform for genetics service delivery. *BMJ Open* 2022;**12**(4):e060899.

For peer review only

37. Bombard Y, Clausen M, Shickh S, et al. Effectiveness of the Genomics ADvISER decision aid for the selection of secondary findings from genomic sequencing: a randomized clinical trial. *Genetics in Medicine* 2020;**22**(4):727-35.
38. Bombard Y, Clausen M, Mighton C, et al. The Genomics ADvISER: development and usability testing of a decision aid for the selection of incidental sequencing results. *European Journal of Human Genetics* 2018;**26**(7):984-95.
39. Lawrence CE, Dunkel L, McEver M, et al. A REDCap-based model for electronic consent (eConsent): Moving toward a more personalized consent. *Journal of Clinical and Translational Science* 2020;**4**(4):345-53.
40. Kirk EP, Ong R, Boggs K, et al. Gene selection for the Australian Reproductive Genetic Carrier Screening Project ("Mackenzie's Mission"). *Eur J Hum Genet* 2021;**29**(1):79-87.
41. Ceyhan-Birsoy O, Machini K, Lebo MS, et al. A curated gene list for reporting results of newborn genomic sequencing. *Genet Med* 2017;**19**(7):809-18.
42. Milko LV, O'Daniel JM, DeCristo DM, et al. An Age-Based Framework for Evaluating Genome-Scale Sequencing Results in Newborn Screening. *J Pediatr* 2019;**209**:68-76.
43. Martin AR, Williams E, Foulger RE, et al. PanelApp crowdsources expert knowledge to establish consensus diagnostic gene panels. *Nature Genetics* 2019;**51**(11):1560-65.
44. Stark Z, Foulger RE, Williams E, et al. Scaling national and international improvement in virtual gene panel curation via a collaborative approach to discordance resolution. *The American Journal of Human Genetics* 2021;**108**(9):1551-57.
45. Richards S, Aziz N, Bale S, et al. Standards and guidelines for the interpretation of sequence variants: a joint consensus recommendation of the American College of Medical Genetics and Genomics and the Association for Molecular Pathology. *Genetics in Medicine* 2015;**17**(5):405-23.
46. Murray MF, Giovanni MA, Doyle DL, et al. DNA-based screening and population health: a points to consider statement for programs and sponsoring organizations from the American College of Medical Genetics and Genomics (ACMG). *Genet Med* 2021;**23**(6):989-95.
47. Marteau TM, Bekker H. The development of a six-item short-form of the state scale of the Spielberger State—Trait Anxiety Inventory (STAI). *British Journal of Clinical Psychology* 1992;**31**(3):301-06.
48. Van Zuuren FJ, De Groot KI, Mulder NL, et al. Coping with medical threat: An evaluation of the Threatening Medical Situations Inventory (TMSI). *Personality and Individual Differences* 1996;**21**(1):21-31.
49. Ong LM, Visser MR, van Zuuren FJ, et al. Cancer patients' coping styles and doctor-patient communication. *Psychooncology* 1999;**8**(2):155-66.
50. Wakefield CE, Homewood J, Mahmut M, et al. Usefulness of the Threatening Medical Situations Inventory in individuals considering genetic testing for cancer risk. *Patient Educ Couns* 2007;**69**(1-3):29-38.
51. O'Connor AM. Validation of a decisional conflict scale. *Med Decis Making* 1995;**15**(1):25-30.
52. Zellerino B, Milligan SA, Brooks R, et al. Development, testing, and validation of a patient satisfaction questionnaire for use in the clinical genetics setting. *Am J Med Genet C Semin Med Genet* 2009;**151c**(3):191-9.
53. Brehaut JC, O'Connor AM, Wood TJ, et al. Validation of a decision regret scale. *Med Decis Making* 2003;**23**(4):281-92.

For peer review only

54. Li M, Bennette CS, Amendola LM, et al. The Feelings About genomiC Testing Results (FACToR) Questionnaire: Development and Preliminary Validation. *J Genet Couns* 2019;**28**(2):477-90.
55. Grant PE, Pampaka M, Payne K, et al. Developing a short-form of the Genetic Counselling Outcome Scale: The Genomics Outcome Scale. *Eur J Med Genet* 2019;**62**(5):324-34.
56. Weiner BJ, Lewis CC, Stanick C, et al. Psychometric assessment of three newly developed implementation outcome measures. *Implementation Science* 2017;**12**(1):108.
57. Proctor E, Silmere H, Raghavan R, et al. Outcomes for implementation research: conceptual distinctions, measurement challenges, and research agenda. *Adm Policy Ment Health* 2011;**38**(2):65-76.

For peer review only

Figure 1: Recruitment flow diagram including survey time points. Microphone denotes timing of appointment recordings. PIS: patient information sheet; GC: genetic counsellor; AF: additional findings

Figure 2: Visual aide for use during pre-test genetic counselling appointment detailing the different types of additional findings

For peer review only

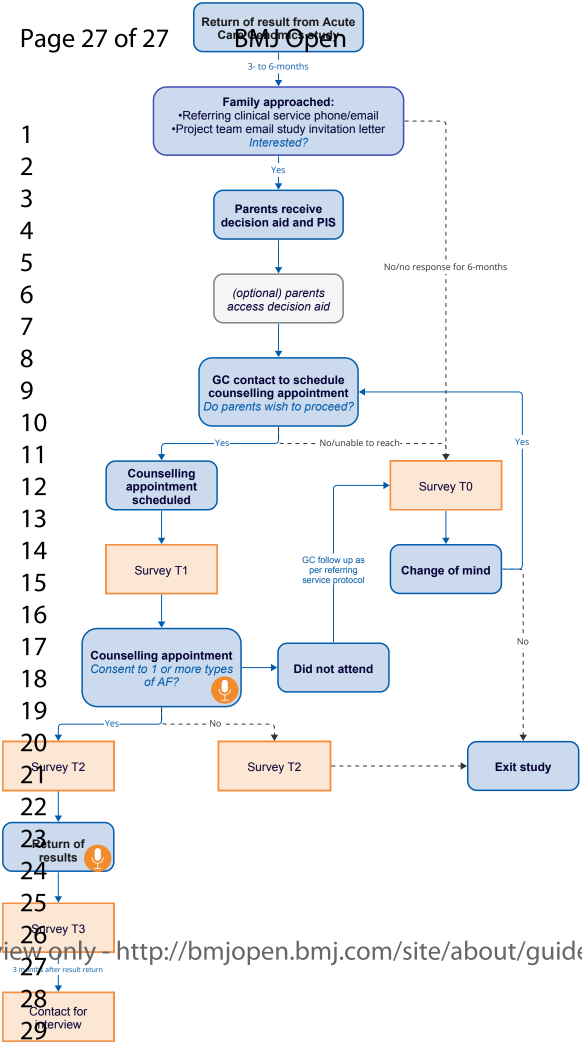

view only - http://bmjopen.bmj.com/site/about/guide

Types of Information Offered

	Childhood-onset Additional Findings	Adult-onset Additional Findings	Genetic Carrier Screening
**Who is the information about?**	Your child	Both parents separately	Both parents together
**What information is being looked for?**	Conditions that affect children. These may or may not have a known treatment or intervention to improve your child's health	Conditions that may develop in adulthood with a known treatment or intervention	Increased chance as a couple of having a child with a different severe or life-threatening genetic condition. Some are treatable, others are not
**Examples**	Muscular dystrophy, some types of blindness or deafness	Hereditary breast and ovarian cancer, hereditary bowel cancer, genetic heart conditions	Carrier for cystic fibrosis, sickle cell disease, some forms of developmental delay
**Impact**	Can reveal your child's chance of developing another genetic condition	You may need more tests and procedures	Can impact pregnancy planning

For peer review only - http://bmjopen.bmj.com/site/about/guidelines.xhtml
